# Prioritizing causal disease genes using unbiased genomic features

**DOI:** 10.1186/s13059-014-0534-8

**Published:** 2014-12-03

**Authors:** Rahul C Deo, Gabriel Musso, Murat Tasan, Paul Tang, Annie Poon, Christiana Yuan, Janine F Felix, Ramachandran S Vasan, Rameen Beroukhim, Teresa De Marco, Pui-Yan Kwok, Calum A MacRae, Frederick P Roth

**Affiliations:** Cardiovascular Research Institute, University of California, San Francisco, CA 94158 USA; Department of Medicine, University of California, San Francisco, CA 94143 USA; Institute for Human Genetics, University of California, San Francisco, CA 94158 USA; California Institute for Quantitative Biosciences, San Francisco, CA 94143 USA; Department of Biological Chemistry and Molecular Pharmacology, Harvard Medical School, Boston, MA 02115 USA; Department of Medicine, Brigham and Women’s Hospital, Harvard Medical School, Boston, MA 02115 USA; Donnelly Centre and Departments of Molecular Genetics and Computer Science, University of Toronto and Lunenfeld Research Institute, Mt Sinai Hospital, Toronto, Ontario M5G 1X5 Canada; Department of Epidemiology, Erasmus University Medical Center, PO Box 2040, 3000 CA Rotterdam, The Netherlands; Preventive Medicine and Cardiology Sections, and Department of Medicine, Boston University School of Medicine, Boston, MA 02118 USA; Framingham Heart Study, Boston University School of Medicine, Framingham, MA 01702 USA; Center for Cancer Genome Discovery and Department of Cancer Biology, Dana-Farber Cancer Institute, Boston, MA 02215 USA; Center for Cancer Systems Biology (CCSB) and Department of Cancer Biology, Dana-Farber Cancer Institute, Boston, MA 02215 USA; The Canadian Institute for Advanced Research, Toronto, ON M5G 1Z8 Canada

## Abstract

**Background:**

Cardiovascular disease (CVD) is the leading cause of death in the developed world. Human genetic studies, including genome-wide sequencing and SNP-array approaches, promise to reveal disease genes and mechanisms representing new therapeutic targets. In practice, however, identification of the actual genes contributing to disease pathogenesis has lagged behind identification of associated loci, thus limiting the clinical benefits.

**Results:**

To aid in localizing causal genes, we develop a machine learning approach, *O*bjective *P*rioritization for *E*nhanced *N*ovelty (OPEN), which quantitatively prioritizes gene-disease associations based on a diverse group of genomic features. This approach uses only unbiased predictive features and thus is not hampered by a preference towards previously well-characterized genes. We demonstrate success in identifying genetic determinants for CVD-related traits, including cholesterol levels, blood pressure, and conduction system and cardiomyopathy phenotypes. Using OPEN, we prioritize genes, including *FLNC*, for association with increased left ventricular diameter, which is a defining feature of a prevalent cardiovascular disorder, dilated cardiomyopathy or DCM. Using a zebrafish model, we experimentally validate *FLNC* and identify a novel *FLNC* splice-site mutation in a patient with severe DCM.

**Conclusion:**

Our approach stands to assist interpretation of large-scale genetic studies without compromising their fundamentally unbiased nature.

**Electronic supplementary material:**

The online version of this article (doi:10.1186/s13059-014-0534-8) contains supplementary material, which is available to authorized users.

## Background

Genetic variant discovery offers two potential clinical benefits: improved estimation of disease risk for individual patients, and identification of novel therapeutic targets [1]. Although the predictive utility of common disease variants has been modest, unexpected connections between genes and diseases, such as the role of the complement pathway in age-related macular degeneration [2], have emerged to steer treatment strategies in promising, unforeseen directions [3].

Genetic studies can contribute to target discovery only if the genes underlying the genotype-phenotype relationship can be identified unambiguously. In the case of genome-wide association (GWA) studies, the associated single nucleotide polymorphism (SNP) is typically only a proxy for a ‘block’ of variants with closely correlated genotypes, so that the associated SNP is often termed a ‘tag SNP’. The associated blocks can be separated by many tens of kilobases (kb), and can overlap dozens of genes [4], any one of which might harbor the causal variant. The interpretation of sequencing-based studies of human disease has also been challenging: the thousands of potentially deleterious mutations in the human genome have made it difficult to pinpoint the actual causal gene(s) [5].

To resolve this ambiguity, investigators have turned to 'pathway' approaches, where increased confidence in the causality of genes is derived from genes at multiple loci sharing some functional aspects [6]. Although such approaches have helped identify some commonality amongst genes at implicated loci, such as the importance of the skeletal system in determining height [7], they typically rely in whole or in part on biased, investigator-driven gene annotation - for example, Gene Ontology (GO) terms [8-11], protein-protein interactions from studies targeting specific proteins of interest [12,13], or published abstract co-occurrence [14]. This presents a paradox - despite starting from unbiased genome-wide data, such pathway approaches tend to identify only well-studied genes.

Genetic research in cardiovascular disease (CVD) has fit the general pattern described above. Our understanding of both Mendelian and complex forms of CVD has benefited from the application of genome-wide technologies, with hundreds of loci implicated in such important disease traits as cholesterol and triglyceride level [15], cardiac conduction phenotypes [16-20], and disorders of cardiac muscle [21,22]. Nonetheless, progression from this vast catalog of data towards actual therapeutic targets has been hampered by the inability to separate causal genes from bystanders.

As an aid to interpreting large-scale association and sequencing data in CVD, we have developed a strategy we term Objective Prioritization for Enhanced Novelty (OPEN), a machine learning approach that prioritizes causal genes based entirely on the sharing of unbiased genomic features. Our approach uses either GWA loci or Mendelian disease genes as a source of positive training examples, and derives a predictive model that can be applied to score all genes in the genome for likelihood of disease association. Using publicly available databases, we compiled >40,000 genomic features capturing diverse gene characteristics, none of which would be expected to favor well-studied genes. We assessed our method on a variety of cardiac traits using cross-validation and observed strong performance. Furthermore, with the help of OPEN-prioritized gene-disease associations from a GWA study on left ventricular dimension, we identified promising candidate causal genes that would otherwise have failed genome-wide significance criteria. Three of these genes (*SVIL*, *FLNC*, *USP13*) were validated in a zebrafish model of cardiac function. Finally, we sequenced the exons of these prioritized candidates in patients with idiopathic dilated cardiomyopathy (DCM), and found one patient harboring a conserved splice-site mutation in the novel cardiomyopathy-associated *FLNC* gene.

## Results

Here we describe 1) an overview of the OPEN approach, 2) assembly of unbiased genomic features we used, 3) the objective use of prior experimental data to weight training examples, 4) details of the OPEN algorithm, and 5) an evaluation of performance, through cross-validation, experimental follow-up, and genetic sequencing of novel candidate genes in patients affected with CVD.

### The OPEN strategy for prediction of causal genes

**Figure 1:**
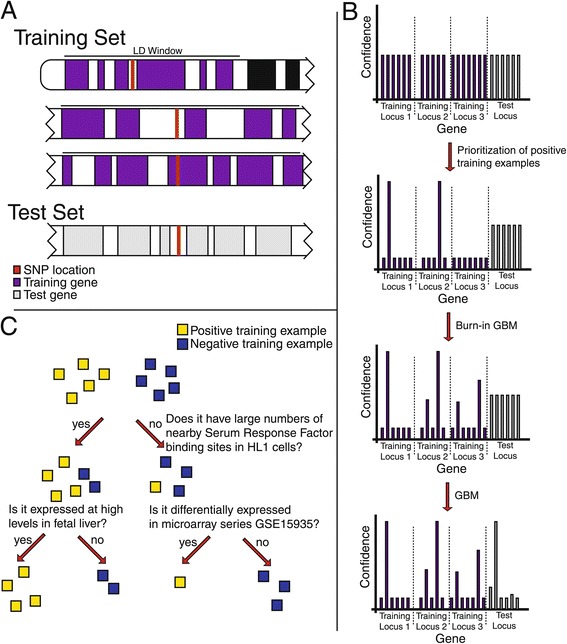
**A decision tree-based approach for causal gene prediction. (A)** Mapping of SNPs to neighboring genes using a combination of linkage disequilibrium (LD) information and the location of recombination hotspots. **(B)** Workflow applying OPEN for causal gene prediction at GWA loci. GWA loci are represented by horizontal bars with individual genes represented by vertical bars. The bar height represents the probability that a gene is causal for the phenotype of interest. Initially, all probabilities are equal. Probabilities are then preliminarily updated based on physical distance from index variant or, optionally, if any prior experimental evidence links them to the phenotype of interest. These probabilities are used in the sampling of positive training examples at each locus during the construction of decision trees. After a 'burn-in' phase, only genes meeting a probability threshold are used as positive training examples. Through cross-validation, the output of the analysis is the log-odds of disease association for all genes in the genome. GBM, gradient boosting machine. **(C)** Representation of a sample decision tree used for partitioning positive and negative training examples. A classifier consists of multiple decision trees combined in an additive manner.

To perform OPEN, we start with a list of genomic loci associated with a disease of interest, each represented by a single tag SNP (Figure 1), or, in the case of Mendelian disease, a list of genes previously implicated by linkage or whole exome analysis. For GWA, we initially map each tag SNP to neighboring genes in two steps. First, we identify an associated ‘block’ of SNPs in linkage disequilibrium with the tag SNP (using a threshold r^2^ value of 0.5). Second, we identify all genes overlapping this block (Figure 1A; Materials and methods). To account for the fact that enhancers may act at a great distance, we define genes by an inclusive interval extending 250 kbp to either side of a transcription start site. SNPs that reside within the gene body, as defined by transcription start and stop sites, are also assigned to the corresponding gene. Additional SNPs are included on the basis of linkage disequilibrium and nucleotide distance. For each phenotype we identify loci containing tagSNPs, and the gene(s) overlapping these loci represent our initial positive training examples. At each locus, we then apply a weight to each gene that reflects proximity to the tag SNP as well as prior experimental evidence that implicates it in a relevant biological process. Such evidence might include association with a Mendelian form of the disease, an ortholog in a mouse model that exhibits a cognate phenotype, or annotation with a GO term that is held by other genes associated with the disease of interest. We then apply two rounds of machine learning. The purpose of the first round is to limit and refine the list of training examples from among genes at disease-associated loci, while the second round aims to score candidate genes according to likelihood of disease association. The output of the first round is a reduced subset of positive training examples that stand out relative to their peers at each locus (see Materials and methods). Genes are not selected from loci which contain many genes unless they are high-scoring outliers, while genes at sparse loci (loci containing a small number of genes within the linkage disequilibrium window) have a high likelihood of being included. The second round uses this enriched set of positive examples for training to derive a predictive model of disease association. For predictions related to Mendelian disease association, where there is no ambiguity in SNP-to-gene mapping, the first round is skipped, and all training examples are retained for the second round.

Note that, like all other disease gene prioritization strategies, our positive training sets are derived through use of the literature and other potentially biased information sources. However, OPEN frees itself completely from investigation bias in its use of unbiased genomic features to assess similarity among genes and thereby exploit these refined positive training examples to make new predictions.

### Genomic features

In principle, any gene feature expected to be held (or avoided) in concert for disease genes can be used in this approach. However, given our concern for biasing predictions towards well-studied genes, we built an extensive set of >40,000 features with minimal preference for previously characterized genes. Genomic features were derived from publicly available genome-wide expression data, transcription factor binding information (both observed and predicted), phylogenetic profiles, protein domain organization, and predicted microRNA (miRNA) targets (Table 1). In most cases, preprocessing was required to generate useful predictors. For example, for microarray analysis, we automated downloading, normalization, clustering, and differential gene expression analysis for 1,437 human and murine microarray data sets obtained through the Gene Expression Omnibus (GEO) [23]. As a comparison to assess the impact of biased features on our predictions, we also downloaded GO annotations and used each of the 17,156 terms as descriptive features.

**Table 1:** **Genomic features used in OPEN**

**Data type**	**Data source**	**Number of features**
Differential mRNA expression	GEO - human	15,778
Differential mRNA expression	GEO - mouse	4,780
Tissue mRNA expression - percentile	Novartis Tissue Atlas	158
Tissue mRNA expression - percentile	Neurocrine Tissue Atlas	2,031
Tissue mRNA expression - comparison	Neurocrine Tissue Atlas	141
mRNA expression clusters	Liver	148
mRNA expression clusters	Multi-tissue	108
Protein domain organization	Interpro	12,624
Predicted conserved TFBS	Vista	2,336
Chip-Seq	Encode/GEO	2,073
Predicted TFBS	PWM (Jasper/Transfac/Uniprobe)	12,624
Predicted conserved TFBS	Vista	2,336
miRNA predicted binding sites	TargetScan	153
Phylogenetic profiles	Ensembl	49

GEO, Gene Expression Omnibus; PWM, position weight matrix; TFBS, transcription factor binding site.

### Principled prioritization

A recurring message in the interpretation of GWA data for many traits is that, for a (sometimes small) subset of associated loci, the likely causal gene is obvious from prior experimental work. Such evidence might include linkage studies of a corresponding Mendelian disease or might have emerged from the thousands of mouse genetic models previously generated. In the case of lipid profiles, for example, 16 of 21 genes involved in Mendelian forms of cholesterol or triglyceride disorders (Additional file 1) also harbor common variants significantly associated with cholesterol/triglyceride levels in GWA studies. Similarly, at least 10% of loci implicated in height contain at least one gene that had been previously implicated in skeletal morphology [7]. We sought to apply this information systematically as a source for weighting individual gene candidates within GWA loci, upweighting specific training examples that were also causally mutated for a corresponding Mendelian disease or that share GO or mouse phenotype annotation with genes from other associated loci (Materials and methods; Additional file 2). We also used a more stringently defined set of these upweighted training examples as 'likely positives' for performance metrics (see below).

### An adaptation of the gradient boosting algorithm for analyzing genetic loci

In principle, the OPEN approach is compatible with a number of machine learning algorithms. We selected and adapted the gradient boosting machine (GBM) algorithm [24], in part because of its suitability for learning problems where only a small fraction of features examined are informative [25], and because of the ability to perform stochastic sampling of training examples, which is well suited to the problem of finding the most likely gene(s) explaining the association signal at GWA loci. GBM, which was a critical component of the winning 'Netflix Grand Prize' solution [26,27], has been used for a variety of biological and medical machine learning problems, including deciphering the tissue-specific splicing code [28] and predicting clinical outcomes in osteoporosis [29]. GBM builds an additive expansion of small decision trees (Figure 1B), with each tree partitioning genes based on a series of informative features differentiating positive and negative training examples. Boosting is used so that successive decision trees focus on classifying those examples that had been poorly differentiated up to that point. Stochasticity is introduced by subsampling positive and negative training examples for each tree.

Machine learning using positive training examples from GWA loci presents a particular problem because of the organization of genes in the genome - specifically the fact that genome often harbors clusters of contiguous functionally related paralogs. Extreme examples include the olfactory gene cluster at the 11p15.4 region, the histone cluster at 6p22.1, and the interleukin receptor cluster at 2q12. If one naively put all genes at all associated loci in a 'bag' and looked for shared features, one could identify a feature common to dozens of paralogous genes within one locus that is not shared by genes within any other locus. Under the assumption that one or perhaps only a few genes underlie the GWA signal at any locus, it is essential to find features shared across loci rather than within loci. Our stochastic sampling scheme thus sampled only a single gene at each locus, with probability of sampling given by its weight.

### Performance evaluation

**Figure 2:**
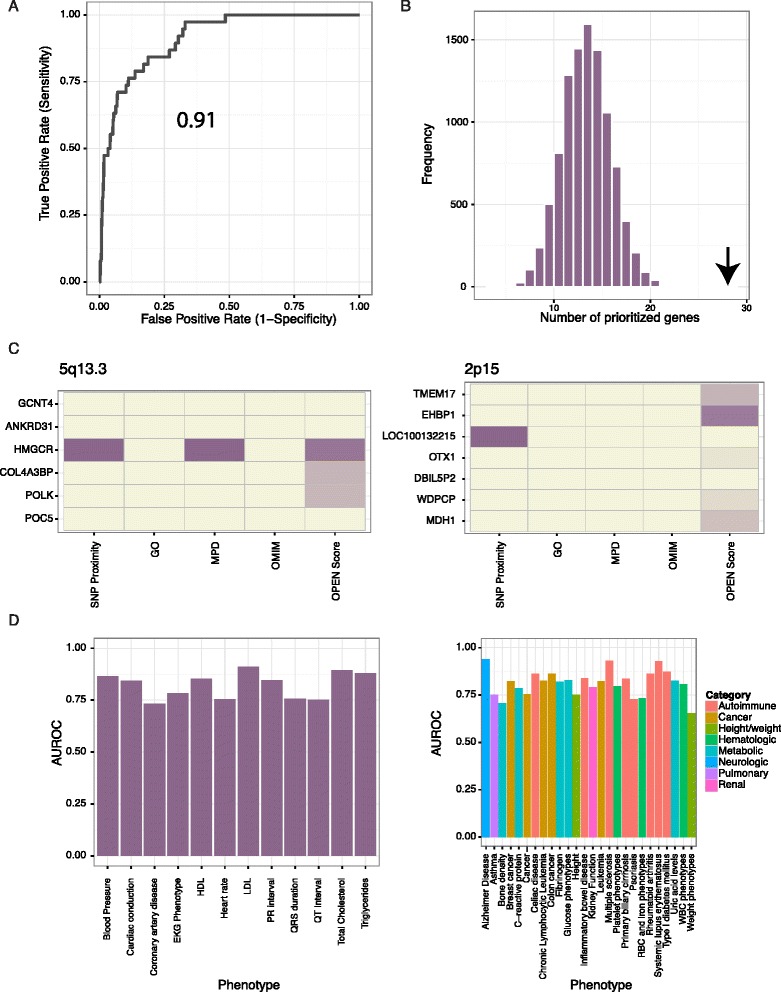
**OPEN successfully prioritizes causal genes for complex traits. (A)** Receiver operating characteristic (ROC) curves for prioritization of 'likely positive' genes for LDL-cholesterol. **(B)** OPEN effectively prioritizes likely positives for low-density lipoprotein (LDL)-cholesterol within GWA loci. A histogram shows the distribution of the number of genes prioritized by random chance over 10,000 independent simulations, with arrow indicating the number prioritized by OPEN (*P* < 0.0001). **(C)** OPEN successfully prioritizes the statin target *HMGCR* at the 5q13.3 locus (left). A heatmap depicts the six genes at the LDL-associated 5q13.3 locus, with the first four columns indicating which genes are near the index variant, and which have been annotated with prior evidence via the Gene Ontology (GO), Mouse Phenotype Database (MPD) or through the Online Mendelian Inheritance in Man (OMIM) database. The final column depicts the OPEN score, with color scheme from beige to dark purple indicating increasing magnitude of the log odds for disease association provided by OPEN. At the 2p15 LDL-associated locus (right), OPEN ranks the un-annotated *EHBP1* gene highest. **(D)** Area under the ROC curve (AUROC) values for cardiac (left) and non-cardiac phenotypes (right). EKG, electrocardiogram; HDL, high-density lipoprotein.

Cross-validation was used to estimate the odds of disease association for every gene in the genome. We initially focused on CVD complex trait phenotypes for which GWA studies had identified 10 or more significantly associated loci (Figure 2A-D). These included plasma concentrations of cholesterol subfractions (high-density lipoprotein-cholesterol (HDL-C), low-density lipoprotein (LDL)-cholesterol, total cholesterol), plasma triglycerides, hypertension/blood pressure, heart rate, and three electrocardiographic phenotypes (QRS duration, QT interval, PR interval, as well as pooled phenotypes of all of these).

The primary use of our causal gene prioritization is for the correct ordering of a short list of genes, either within a locus found through a GWA study or obtained through filter-based variant prioritization for gene sequencing studies. We therefore evaluated the results in three ways compatible with these goals. First we used the area under the receiver operating characteristic curve (AUROC), which assesses the probability of successfully discriminating a randomly chosen positive training example from a random negative example. Second, we used a permutation-based assessment of the chance that 'likely positives' would rank near the top of the list of genes at each disease locus (Materials and methods). This metric most closely coincides with the needs of biologists pursuing GWA signals, pedigree-based linkage peaks or filtered lists of exome sequencing variants, where the goal is to identify the most likely candidate among a limited subset of genes. Finally, we used precision (fraction of predictions that are correct, also known as positive predictive value) at specific levels of sensitivity to assess whether an experimentalist who did not have access to genetic data and instead proceeded down the list of predictions validating each one by one would find a high percentage of successes.

Focusing on serum LDL-cholesterol as a phenotype, cross-validation showed an AUROC of 0.91, indicating excellent performance (an AUROC of 1 represents perfect classification, while a value of 0.5 is no better than random chance; Figure 2A). Moreover, we were able to prioritize 28 of the 38 total 'likely positive' genes at or near the top of their respective loci (*P* < 0.0001, Fisher’s exact test; Figure 2B,C). For example, at the 5q13.3 locus we prioritized the statin target *HMGCR* as the most likely causal gene, despite only using unbiased genomic features as predictors. Similarly, at the 2p15 locus, we prioritized *EHBP1*, which was recently shown to be associated with the Mendelian LDL-cholesterol gene and therapeutic target *PCSK9* in murine and cellular models [30]. Overall, performance figures for other cardiac phenotypes were also excellent, with AUROC values ranging between 0.75 and 0.9 (Additional file 3). Repeating the analysis of cardiac phenotypes without the use of prior information to up-weight training genes resulted in only a moderate drop in performance (for example, from 0.85 to 0.80 for triglycerides, from 0.84 to 0.78 for cardiac conduction phenotypes). This suggests that while prior evidence is certainly an asset for prediction, it does not appear to be a requirement.

Performance estimates based on genome-wide precision or positive predictive value of highly ranked genes were not as strong as AUROC results for classification within implicated loci (Additional file 3). The precision (positive predictive value) at 20% recall (sensitivity) ranged from 2 to 6% for lipid profile traits, and lower for other traits. While 2 to 6% precision is far better than would have been expected by chance (for example 0.1% is expected for plasma triglycerides, *P* = 6.6 × 10^-5^), whether it is sufficient to justify sequential experimental validation of targets depends on the value of identifying new causal genes relative to the expense of the experimental validation effort. Investigators may also have additional orthogonal evidence that might be used to further enrich positives within this prioritized list, allowing a pinpointing of causal genes.

### Applying OPEN to non-cardiac traits

Given the breadth of our genomic features, OPEN should be also effective in pinpointing causal genes across non-cardiac phenotypes. We therefore extended our approach to 26 additional non-cardiac traits, including autoimmune, hematologic, metabolic and cancer phenotypes, and observed strong success in prioritizing causal genes (Additional files 3, 4, 5, 6, 7, 8, 9, 10, 11, 12, 13, 14, 15, 16, 17, 18, 19, 20, 21, 22, 23, 24, 25, 26, 27, 28, 29, 30, 31, 32, 33, 34, 35, 36, 37, 38, 39, 40 and 41). For example, we successfully prioritize 27 of 35 likely positives involved in type 2 diabetes and glucose phenotypes (*P* < 0.0001) with an AUROC of 0.83. Thus, OPEN generalizes well to non-cardiac traits.

### Applying OPEN to the heritable cardiomyopathies

In addition to complex types of CVD, there exist a number of monogenic forms, which, in aggregate, are significant contributors to cardiovascular morbidity and mortality. The most prevalent form of monogenic CVD are the cardiomyopathies (CMPs), which are debilitating, frequently fatal diseases of the heart muscle, with a strong heritable component [31]. CMPs have historically been classified on the basis of anatomic and physiologic characteristics as belonging to hypertrophic (HCM), dilated (DCM) or restrictive subtypes. Genetic linkage studies have identified a number of disease genes underlying HCM and DCM. Given that the first presentation of CMP can be sudden cardiac death, knowledge of such genes has been of considerable value in clinical care, as it allows genetic screening of family members, resulting in careful surveillance of mutation-carrying but asymptomatic individuals and early implantation of life-saving defibrillator therapy.

**Figure 3:**
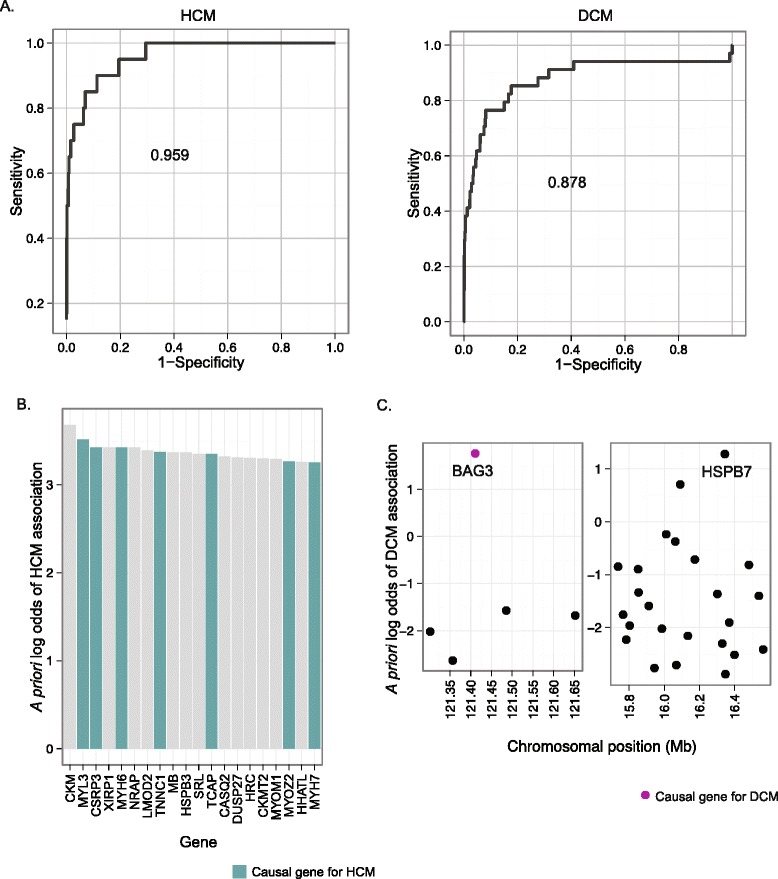
**OPEN successfully predicts cardiomyopathy genes. (A)** ROC curves for hypertrophic cardiomyopathy (HCM, left) and dilated cardiomyopathy (DCM, right). **(B)** Top ranked genes according to OPEN score for HCM. Log-odds of disease association are obtained through cross-validation. Blue bars represent positive training examples. **(C)** OPEN scores for DCM (as a Mendelian disease) are useful for prioritizing genes at DCM GWA loci. Each locus is represented by a scatter plot of OPEN score against chromosomal position, with every gene at the locus represented by a circle. Gene symbols for top ranked genes are provided. Purple coloring indicates that *BAG3* has already been implicated in a Mendelian form of DCM.

Given that only 25 to 60% of familial cases of DCM or HCM can be explained by our current census of CMP genes [32,33], genetic studies of CMP patients remain a priority. Unfortunately, GWA results have been difficult to interpret, as causal genes are hard to differentiate amongst the many genes harboring rare mutations [34]. To provide an unbiased, independent method of prioritizing causal CMP genes, we applied our OPEN approach to HCM and DCM, using established causal genes as training examples. OPEN successfully predicted established CMP genes, as evidenced by an AUROC of 0.88 and 0.96 for DCM and HCM, respectively (Figure 3A). The prominent clustering of known CMP causal genes at the top of the list (Figure 3B; precision at 20% recall of 19% for DCM and 42% for HCM) strongly suggests that OPEN scores could be integrated with sequencing data to choose candidates for experimental validation.

### Interpreting GWA study data using Mendelian disease predictions

We hypothesized that OPEN scores for Mendelian diseases such as the CMPs could also be useful for interpreting GWA study results for related phenotypes. For example, a GWA study of DCM identified two loci at genome-wide significance [22]. Overlaying OPEN DCM scores for each of the genes at these loci revealed two promising candidates (Figure 3C): *BAG3*, a recently identified causal DCM gene [35], and *HSBP7*, a gene recently implicated in protection against tachycardia-induced CMP [36] and that also caused a reduction in cardiac output upon disruption in zebrafish [11].

**Figure 4:**
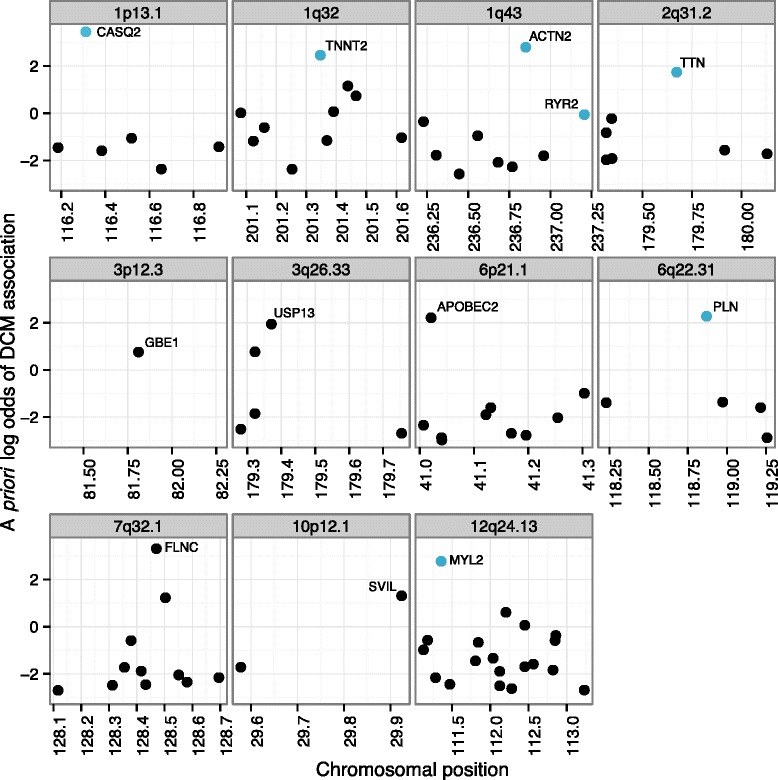
**OPEN scores for DCM successfully prioritize genes at loci identified through a GWA study for left ventricular diameter (LVD).** OPEN scores for DCM were mapped to genes at loci marginally associated with LVD (*P* < 5 × 10^-5^). Eleven loci have a high-scoring top-ranked gene based on OPEN scores for DCM association. Blue coloring indicates the seven genes are mutated in Mendelian forms of cardiac disease, including DCM, HCM, arrhythmogenic right ventricular cardiomyopathy and catecholaminergic polymorphic ventricular tachycardia (*P* = 8 × 10^-5^ for enrichment).

Left ventricular diameter (LVD) is a heritable complex trait that predicts incident congestive heart failure and mortality [37]. A recent GWA meta-analysis of LVD found only a single locus at genome-wide significance [21], though interestingly, the locus includes *PLN* (which encodes phospholamban), a known DCM gene [38]. Given the phenotypic similarity between DCM and enlarged LVD, we hypothesized that OPEN scores for DCM would be useful to prioritize genes at LVD loci, even if these failed to meet genome-wide significance in GWA analysis. We first selected all SNPs with a nominal *P*-value of 5 × 10^-5^ (considerably less stringent than the conventionally accepted threshold of 5 × 10^-8^ for significance), mapped these to neighboring genes, and looked for any loci where the top-ranked gene (according to DCM score) had a higher score than that expected based on random chance (see Materials and methods). We found 11 such loci (of which 10 were multigenic; Figure 4). For four of these loci, the top gene according to OPEN had been shown to be mutated in DCM (*PLN*, *ACTN2*, *TNNT2*, *TTN*) [39], while for two others, the top gene was known to be mutated in HCM (*MYL2*) or an arrhythmogenic form of cardiac disease (*CASQ2*) [40]. For two more, the top gene (*GBE1* and *APOBEC2*) affects heart contractile function in animal models [41,42]. Note that the OPEN procedure is ‘clean’, in the sense that the score for any given gene is derived from models that never used that gene in training. Overall, the 'insignificant' LVD GWA loci were highly enriched for genes causing inherited abnormalities of the heart muscle (*P* = 8 × 10^-5^, odds ratio = 7.65, Fisher’s exact test).

### Validating prioritized candidates in zebrafish

**Figure 5:**
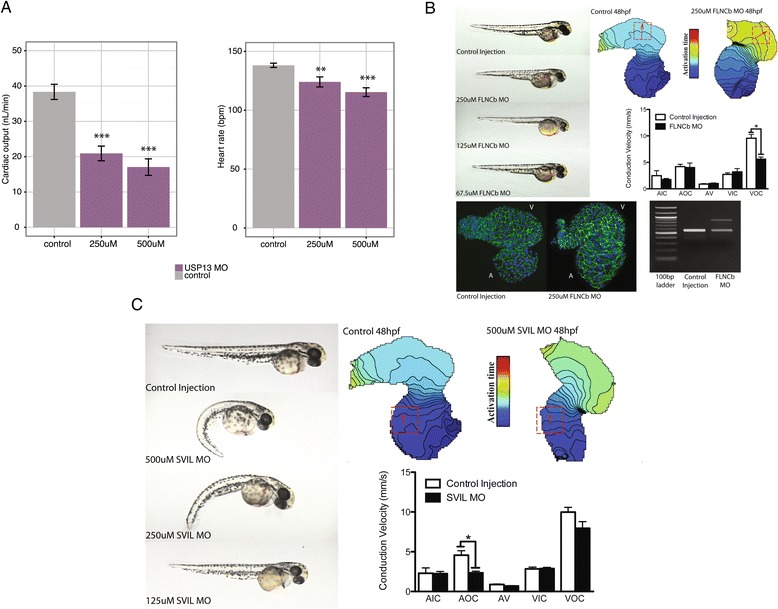
**OPEN prioritized genes contribute to cardiac phenotypes in zebrafish. (A)** Knockdown of *USP13* caused a dose-dependent decrease in cardiac output, due to both a decrease in heart rate and ventricular stroke volume. **(B)** Injection of a morpholino (MO) targeting a specific splicing event in *FLNCb* (see Materials and methods) caused apparent cardiac-specific defects. Images on the right show embryos at 48 hours post-fertilization (hpf) with decreasing injected morpholino concentration. Optical mapping confirmed a significant decrease in cardiac conduction velocity in isolated hearts following *FLNCb* splice inhibition (top right: isochronal maps on right, red box indicates measured region of interest, isochrones are 5 ms apart). Conduction velocity was unaltered in other regions of the heart (middle right: bar graph, regions examined were atrial inner curvature (AIC), atrial outer curvature (AOC), AV node (AV), ventricular inner curvature (VIC), and ventricular outer curvature (VOC)). Additionally, *FLNCb* splice inhibition resulted in increased atrial cardiomyocyte size (bottom left: beta-catenin stained in green, DAPI in blue, V and A denote ventricle and atria, respectively). RT-PCR confirmed inhibition of the predicted splicing event in *FLNCb* (bottom right). **(C)** Knockdown of *SVIL* causes cardiac edema as well as noticeable spinal curvature at higher morhpolino doses, with only cardiac edema notable at lower doses. Images on left again show decreasing morpholino dose at 48 hpf. Optical mapping (right) confirmed a significant decrease in atrial conduction velocity following SVIL knockdown. ***P < 0.001, **P < 0.01, *P < 0.05.

We focused on the remaining candidates (*USP13*, *FLNC*, *SVIL*), which, at the time of our study, had no known link to cardiac function but were attractive given that: 1) they were found in loci that had been associated with LVD; and 2) they had high OPEN scores for DCM despite having a small number of genes at their locus. All three ranked highly on our overall genome-wide ranking of DCM candidates with *FLNC* being the number 2 candidate overall, USP13 number 105, and SVIL number 298. We knocked each of these down in embryonic zebrafish and looked for evidence of phenotypic change. All three demonstrated abnormalities in cardiac morphology or function (Figure 5). Specifically, microinjection of a morpholino targeting *USP13* caused a dose-dependent decrease in cardiac output. Notably, this decrease was due to both decreased ventricular stroke volume and heart rate (Figure 5A). Next, although previous efforts have described skeletal muscle-specific defects following morpholino-based introduction of a premature stop codon in *flncb* (one of two zebrafish orthologs of human *FLNC* [43]), cardiac defects related to *FLNC* have not been explicitly described. Following injection of a splice-blocking morpholino we noted obvious distention of the atrium and backflow upon cardiac contraction (Additional file 42). Immunological staining confirmed hypertrophy of atrial cardiomyocytes (Figure 5B). Additionally, optical mapping showed a decrease in ventricular conduction velocity in isolated hearts following *flncb* splice inhibition compared with sham-injected controls, suggesting alterations in junctional remodeling and cell-cell coupling (Figure 5B). Finally, knockdown of our third candidate, *SVIL*, caused obvious pericardial edema at low morpholino doses (Figure 5C) with optical mapping confirming an underlying decrease in atrial conduction velocity.

To evaluate the extent to which incorporation of biased features may compromise prioritization of previously unstudied genes, we repeated the OPEN predictions for DCM but included as predictive features >17,000 GO categories. We noted that our experimentally validated candidate *USP13* dropped from 105 in our overall ranking of genes in the genome down to 1,614 - which, depending on the validation strategy and budget, would likely have removed it from consideration for experimental follow-up.

### Validation of prioritized candidates through targeted exon sequencing in DCM patients

Given our experimental results, we hypothesized that our prioritized list of DCM candidates may in fact help identify disease-causing variants in actual patients. We designed capture probes corresponding to all known exons of established (34) and candidate (13) DCM genes and genotyped 60 patients with idiopathic DCM using next-generation sequencing at extremely high depth (>700-fold). We focused on mutations with severe deleterious consequences, including nonsense, frame-shift, and canonical splice acceptor/donor disruptions. In addition to finding several truncating mutations in established DCM genes (data not shown), we identified in one patient with severe DCM (left ventricular ejection fraction of 25% and incessant ventricular tachycardia) a novel splice acceptor mutation in *FLNC* (c.3791 - 1 G>C) that is predicted to lead to exclusion of a 53 amino acid exon and result in a frame shift. For this patient, no established DCM genes harbored predicted pathogenic mutations and a survey of more than 7,500 control samples revealed no comparably damaging mutations in *FLNC* (see Materials and methods). Although informed consent issues preclude establishment of segregation at this time, this is, to our knowledge, the first implication of an *FLNC* mutation in a CMP.

## Discussion

Here we have demonstrated a novel approach (OPEN) for unbiased disease-gene prioritization following genetic screening. The OPEN approach is based on the use of an informative collection of unbiased genomic features coupled with a principled strategy towards incorporating prior knowledge in the weighting of training examples. OPEN provided demonstrably high quality predictions for CMPs, but also had excellent performance across multiple disease categories, suggesting that OPEN could readily be expanded to any trait of interest. We then demonstrated the practical utility of our approach through prospective validation of candidate causal cardiovascular disease genes in zebrafish and through genetic sequencing of human cardiac disease patients.

With the discovery of a conserved splice site mutation in *FLNC* in a patient with DCM, we propose a new candidate for diagnostic testing of DCM patients. Since fewer than 50% of DCM patients receive a positive result from genetic testing [44], additional disease genes are of particular value, as positive mutation information can influence intensity of screening and implementation of preventive therapies. Although FLNC had previously been linked to myofibrillar myopathy [45], a disease of the skeletal muscle, it had not been implicated in cardiac disease. Consistent with the behavior of such genes as *BAG3*, *DES*, *TTN*, and *CRYAB*, *FLNC* appears to demonstrate pleiotropy, with varying degrees of skeletal or cardiac manifestations in individual patients.

Interestingly, despite several years of GWA studies, it is still challenging to identify the most likely causal gene within a locus, even after exhaustive experimental validation of multiple candidates [46]. At some loci, such as the 1p36 hypertension locus, a large number of genes appear to play a causal role [47]. This multiplicity of causal genes justifies our strategy of scoring all genes at a locus rather than simply choosing one candidate as the most likely.

While broadly applicable, the OPEN approach does have some notable limitations. First, as in any learning approach, there was generally poorer performance in those cases where training information was sparse. However, even in some cases with an apparent abundance of training information, such as was available for weight phenotypes, performance was poor (AUROC 0.65). This is analogous to what we have seen for phenotype predictions in other organisms [11], as genetic heterogeneity of a particular phenotype or trait can cause ambiguity in the predictions, manifesting as poor performance.

Additionally, precision-recall rates were lower than expected based on comparable AUROC results; however, there are several potential explanations for this observation. First, there is the challenge of knowing the actual true positive for performance evaluation. We have used a deliberately simple heuristic approach to selecting a 'likely positive', but in many cases this may be incorrect, and other genes we prioritize at a given locus may be playing a more substantial causal role. Similarly, there may be false negatives among those genes we have ranked highly that have not (yet) been found within disease-associated loci. Some of these may in fact play a role in the disease process but lack a common variant that regulates their expression substantially in the cohorts that have been studied and thus are not likely to be discovered through GWA. Additionally, our unbiased genomic features may simply be too weak to identify relationships among causal genes that differ widely in terms of pathogenic mechanism. Consequently, as unbiased genome-scale data continue to be generated in disease-relevant mammalian models and cell culture lines, we expect the accuracy of the OPEN approach to increase in turn.

Finally, at this time, we were not able to incorporate expression quantitative trait loci information into our scheme, in part because it is lacking at sufficient scale for the majority of human tissues. Moreover, even with adequate expression quantitative trait loci information, one often fails to pinpoint a single causal gene and can still be left with a list of genes for further prioritization. However, this could in the future serve as one of many potentially valuable orthogonal data types to refine predictions.

## Conclusions

We have described a principled strategy for prioritizing causal disease genes that demonstrates great success in identifying causal genes for a number of CVD-related traits. By virtue of its reliance on unbiased predictive features, our approach should contribute to the discovery of causal genes using the vast amounts of genetic data emerging from genotyping and sequencing technologies. As genetic data continue to emerge from SNP-array and sequencing technologies, developing techniques capable of quickly identifying underlying causal genes will be essential for disease diagnosis and treatment.

## Materials and methods

### Adaptation of the gradient boosting machine algorithm for OPEN

The GBM algorithm [24] uses an additive expansion of decision trees to determine probabilities for objects (genes in this case) belonging to classes (diseases) of interest. The boosting aspect refers to the fact that every successive tree in the expansion focuses on accurately classifying those training examples predicted poorly by the previous trees. In this way, the prediction accuracy is incrementally improved with each successive tree. Stochasticity is introduced by selecting a subset of training examples for each tree. For every tree, at each split point, features are selected to minimize a loss function (absolute loss, in this case) that reflects the difference between the actual values of the training examples (1 for positive training examples and 0 for negative training examples) and the predicted probabilities (between 0 and 1) generated by the ensemble of decision trees to that point. All features are considered for every tree; although this substantially improves performance in situations where the percentage of predictive features is very low, which we expected to be the case for gene-disease predictions, it requires other measures to prevent overfitting. For GBM, overfitting is minimized by: 1) selecting only a subset of training examples per tree; 2) the height of the trees permitted (number of terminal leaves); 3) the weight added to the log-odds output of each tree (a shrinkage parameter); and 4) the number of trees in the expansion. We optimized all parameters for disease class prediction.

We used six rounds of eight-fold cross-validation, and for each round built an additive expansion of sixty small trees, each with three split points (four terminal leaves). Cross-validation involved holding out a fraction of loci from GWA studies, or a fraction of genes for Mendelian diseases. For each tree, stochasticity was introduced by using genes from 70% of loci (or in the case of Mendelian diseases, 70% of genes) to provide positive training examples. Matching numbers of negative training examples were selected. After each tree was completed, we updated the function relating predictive variables to disease class, which could be transformed to provide a log-odds of disease association for every gene.

For GWA-based prediction, we took a probabilistic approach to selecting positive training examples for each tree. Initially, all genes at a locus were assigned a null probability of being the causal gene (represented by a score of 0). We then increased the score by 5 for those genes with a transcription start site within a certain distance from the index variant (50 kb), based on the reasoning that if this proves to be the causal variant, it is more likely to influence expression of neighboring genes. Quantitative models of transcription based on empirical data have documented a substantial contribution of transcription factor binding sites (TFBSs) influencing expression up to 50 kb from the transcription start site [48].

We also explored upweighting the initial score assigned to positive training examples based on prior experimental evidence of involvement in a similar disease or biological process. The majority of prior GWA experiments have demonstrated that multiple loci underlying complex traits include genes already demonstrated to influence a comparable trait, either in model organisms, cell lines, or in Mendelian forms of the disease. We sought to use this information for two purposes: reweighting positive training examples, and performance assessment (see below). For each complex trait studied, we consulted three separate ontologies for related terms: the GO, the Mouse Phenotype Database (MPD), and the Human Phenotype Ontology Database (derived to characterize diseases within the Online Mendelian Inheritance in Man (OMIM) database). To identify GO terms enriched at GWA loci for a given trait, we took the list of genes close to the index variant and submitted these to the Funcassociate server [49]. All GO terms with a multiple hypothesis testing adjusted *P*-value of <0.05 and log odds score of >1 were selected and all genes annotated to those terms and within GWA loci increased in score by 5. We performed a similar approach using phenotype-to-gene mappings derived from MPD. In the small number of cases where no term was significantly enriched at p-adj <0.05 and log odds score >1, we relaxed one or both of these criteria if the annotation and GWA trait appeared consistent from a biological perspective.

Thus, for every gene within a GWA locus, we increased its initial score by 5 for each form of evidence that supported its implication in regulating the phenotype of interest (we did not attempt to optimize the relative weights contributed by each form of evidence). Thus, a gene could have a maximum initial score of 20 (5 points for proximity, and 5 points for implication by OMIM, GO, and MPD) and a minimum score of 0. For loci where there was only a single gene, this gene was given a score of 1. The scores were converted to probabilities by assuming a total probability of 1 at each locus. Genes with a score of 10 or higher were treated as 'likely positives' (Additional file 2) to assess performance of OPEN predictions.

For GWA-based prediction, a 'burn-in' set of 20 trees was used to narrow the number of positive training examples towards a higher confidence set. At every locus, each gene started off with a score between 0 and 20, and a corresponding probability of being the causal gene. For each tree, 70% of loci were selected, and at each locus, a single positive training example was selected based on this probability distribution for causality. Features were selected to differentiate positive and negative training examples, and the log-odds of disease association calculated for each positive training example. The score of the gene at each locus with the highest OPEN-determined log-odds of disease association was then increased by 1 and the process repeated. After 20 trees, the total score for every locus was tallied, and only those genes with scores >25% of this amount were kept as positive training examples. The respective scores of these genes were then fixed and used for probabilistic sampling for the subsequent 60 trees.

For Mendelian disease gene prediction, we did not require similar elaborate measures to narrow positive training examples. Consequently, no 'burn-in' period was used - and 60 trees were built for classification for each of six rounds of eight-fold cross-validation.

### Predictive genomic features used for OPEN

#### Expression microarray

##### *Overview of microarray processing*

Microarray data were analyzed using R/Bioconductor [50]. Raw data were initially normalized by robust multi-array averaging (RMA) [51] using a custom Chip Description File (CDF) from the Michigan Microarray Lab Version 13) [52]. The Michigan Microarray Lab CDF reanalyzes and matches each probe sequence to the most current gene annotation and produces summarized expression values. Differential gene expression was determined using *limma* [53], with a Benjamini-Hochberg [54] adjustment applied for multiple hypothesis testing.

##### *mRNA tissue expression*

Complete datasets corresponding to the GNF Body Atlas [55] and Neurocrine Tissue Atlases [56] were downloaded from GEO (GSE7307). The GNF atlas includes 158 microarrays corresponding to 79 different human tissue types (2 technical replicates). The Neurocrine atlas includes 676 microarrays corresponding to 65 human tissue types or cell lines (normal and/or diseased), from 10 donors (a total of 141 conditions were available - such as 'normal prostate' or 'diseased prostate'). Intensities for the GNF arrays were averaged across replicates. For each tissue atlas, the intensity corresponding to the 75^th^, 90^th^ and 99^th^ percentile across all tissues was identified. Only those probesets mapping uniquely to a single gene were considered, and for those genes mapping to multiple probesets, the probeset with the highest mean expression across all tissues was used. Each combination of percentile threshold and array was included as a single feature, with 1s and 0s assigned to genes whose expression exceeded or fell below the percentile cutoff, respectively. Imputation was performed for any genes not included on the microarrays.

##### *Differential gene expression*

We hypothesized that genes may have distinctive expression in specific tissues, although not necessarily having high abundance. Thus, for each of the 141 tissue or cell line expression datasets mentioned in 2.1.2, we computed a list of genes that were significantly over-expressed relative to all other tissues (false discovery rate <0.05). Each gene list constituted a single feature.

##### *Gene signatures*

We devised an automated approach to determine gene signatures for a large number of microarrays experiments deposited in GEO. First we identified all experiments performed on the two most commonly used microarray platforms: Affymetrix Human Genome U133A Plus 2.0 and Affymetrix Mouse Genome 430 2.0 Array. Next, for each experiment we downloaded all the corresponding microarrays, normalized them, and performed hierarchical clustering of samples using the *hclust* function in the R statistical framework [57]. We defined individual sample groups based on multiple cutpoints along the dendrogram (corresponding to tree heights of 0.975, 0.95, 0.925) and evaluated differentially expressed genes between groups using *limma* [53]. Features consisted of genes that were significantly changed (false discovery rate <0.15) for a given comparison. Separate features were derived for up- and down-regulated genes. We were able to derive features from 1,048 data series on the Affymetrix Human Genome U133A Plus 2.0 platform, and from 389 data series on the Affymetrix Mouse Genome 430 2.0 Array platform.

##### *Model-based clustering*

We performed model-based clustering using the *mclust* package in R to identify genes with correlated expression patterns [58]. Clustering was performed on the Neurocrine tissue atlas (108 total clusters), as well as on a dataset of 427 human liver samples (GSE9588) [59]. Membership in each cluster was used as a single binary feature.

#### Transcription factor binding sites

##### *Evolutionarily conserved transcription factor binding sites*

A list of predicted TFBSs based on evolutionary conservation was downloaded from the ECRbase (Database of Evolutionary Conserved Regions) website [60]. The exact file used was tfbs_ecrs.hg18mm9.v102.txt. In this file, evolutionarily conserved sites were identified and mapped to transcription factors in the TRANSFAC v9.4 database [61]. Chromosomal positions provided by the authors were mapped to Refseq genes within 25 kb, and for each gene the number of each TFBS was tallied. Four discrete features were created for each TFBS, with a feature corresponding to whether a gene had 1, more than 1, more than 2, or more than 3 copies of the TFBS. Thus, a gene with five copies of a given TFBS would have 1s for all the above features while a gene with two copies would have a score of 1, 1, 0 and 0 for the four features, respectively. Since genes vary widely in the number of TFBSs, including these four different features of varying stringency increased the likelihood of having at least one informative feature for every TFBS (that is, not predominantly 1s or 0s for every gene).

##### *Chip-Seq and ChIP-chip transcription factor binding sites*

The results of 691 individual ChIP-chip or ChIP-Seq experiments were downloaded from GEO [62] or the UCSC Genome Browser [63] and used to generate predictive features. Each binding site was mapped to all nearby genes (within 10 kb). Since genes could have one or more nearby binding sites, we determined the number of binding sites corresponding to the 75^th^, 90^th^ and 99^th^ percentile. Each cutoff was used to derive a separate feature.

##### *Predicted transcription factor binding sites*

Genome-wide TFBSs were predicted for 809 position weight matrices (PWMs). The matching of a PWM to a sequence was determined using an entropy-weighted motif match score (MMS) defined as:

$$ MMS=-\frac{{\displaystyle \sum_b{\displaystyle \sum_j{w}_j \log {}_2\left({f}_{bj}/{p}_b\right)}}}{{\displaystyle \sum_j{w}_j}} $$

The MMS involves comparing the observed frequency *f*_*bj*_ of base *b* at each position *j* in the PWM with the background frequency of that base *p*_*b*_ in the genome and summing over the length of the motif. The weight w_j_ at each position in the PWM is a function of the information content:

$$ {w}_j=2-{\displaystyle \sum \log {}_2\left({f}_{bj}\right)} $$

The MMS thus upweights matches at positions where there is less ambiguity as to which base is preferred. We found that this modification resulted in more consistency between binding sites predicted by PWMs generated by different motif finding programs and greater agreement with those predicted using PWMs in publicly available databases (TRANSFAC [61] and JASPAR [64]).

Thresholds for determining the score required for a match were determined separately for each motif by generating the empiric distribution of scores for DNAse accessible regions across a range of tissues. These scores were fit to a three-component mixture model using the *mclust* package [58]. The three components were interpreted as representing 'high confidence', 'intermediate confidence' and 'low confidence' binding, and the lowest value for the 'high confidence' group used as a threshold for binding. Binding sites were mapped to genes as described for 'ChIP-Seq and ChIP-chip TFBSs'.

#### Protein domain composition

Protein domain compositions were extracted from the file protein2ipr.dat downloaded from the Interpro web site [65]. For each protein domain, we generated a single feature corresponding to all proteins with one or more domains of that type. A total of 12,623 protein domains were included as features.

#### Predicted miRNA binding sites

Predicted target sites for 153 miRNAs were downloaded from the TargetScan database [66], with targets for each miRNA used as a single feature.

#### *Phylogenetic profiles*

Phylogenetic profiles, consisting of the presence or absence of human gene orthologs in 49 other species, were downloaded from the Ensembl database [67]. Presence or absence of an ortholog in each species was used as a feature.

#### Phenotype-to-gene mapping

The 'Catalog of Published Genome-wide association Studies' was downloaded from [68]. The catalog reports the top SNPs from >200 GWA study publications, along with *P*-values of association and a phenotype description. We mapped nearly all GWA study phenotypes to an ontology of phenotype(s), based on the Human Phenotype Ontology [69]. Phenotypes with insufficient numbers of training examples were pooled into composite phenotypes (Additional file 43). We selected a *P*-value threshold of 5 × 10^-8^, and mapped all SNPs that met this criterion to the corresponding phenotype. Most phenotypes included multiple SNPs, often from multiple GWA study publications. We then mapped SNPs to genes, broadly following a previously described strategy [14] with some modifications. An interval was defined for each SNP to include all SNPs in the HapMap [70] that were within an r^2^ of 0.5 of the tag SNP. Each interval was then extended out to the next recombination hotspot, which was obtained from the SNP Recomb Hots Track in the UCSC Genome Browser and mapped to hg18 using the *liftover* tool. Given that enhancers can act at considerable distance, we then extended each locus outward an additional 250 kb. All genes whose transcription start site position was included within the SNP interval were then mapped to that SNP (and therefore to the corresponding phenotype). If no gene was found within the SNP interval, the SNP interval was extended 50 kb upstream and downstream of the SNP position and the search for overlapping genes repeated.

For HCM and DCM we started from several recent reviews [39,71,72] of genes implicated through family studies and updated these with any additional examples from the literature and commercial genetic testing panels that had not been considered (Additional file 44).

### Performance assessment

#### ROC curves

Receiver operating characteristic (ROC) curves evaluate the sensitivity and specificity of prioritizing true positives. Although 'true positives' are clear for Mendelian disease prediction, no such list exists for any GWA trait. Instead we used the 'likely positive' gene set, which should not be biased towards our predictions (see the 'Adaptation of the gradient boosting machine algorithm for OPEN' section above). The AUROC and precision-recall statistics were determined using the *ROCR* R package and in-house scripts [11]. Statistical significance of the observed precision at 20% recall was determined by assuming a binomial distribution for the inclusion of 'likely positive' genes within any given subset of genes.

### Permutation approach to assess the significance of gene rankings

#### Prioritization of 'likely positives'

We assessed the significance of prioritization of 'likely positives' by using as our test statistic the number of times a 'likely positive' candidate was found in the top three ranked genes across all loci for a trait. For loci with three genes, we counted the number of instances where a likely positive gene was in the top two, and for loci with two genes, we counted the number of instances of ranking the likely positive gene the higher of the two. To evaluate the significance of this result, we determined the null distribution of the test statistic by permuting the ranks of genes at each loci 10,000 times.

#### High scoring top-ranked genes at LVD loci

To evaluate the statistical significance of top ranked scores at the LVD loci, for each locus mapping to *n* genes, we generated the null distribution of scores by randomly sampling *n* genes from the distribution of scores 10,000 times, and recording the top score for each iteration. The empirical *P*-value was computed by identifying the fraction of iterations with top scores equal to or greater than the observed score.

To compute the enrichment of cardiomyopathy genes among LVD loci (478 genes; Additional file 45), we constructed a list of 48 genes causally mutated in CMPs (Additional file 46) and performed Fisher’s exact test, assuming a 'universe' of 21,626 genes. We found seven genes at the intersection: *ACTN2*, *MYL2*, *PLN*, *RYR2*, *TMEM43*, *TNNT2*, *TTN*. We did not consider *CASQ2*, which is responsible for a primarily arrhythmogenic phenotype in humans, as a success, although it does result in ventricular defects in animal mutants [40].

### Experimental validation of LVD prediction in zebrafish

#### Zebrafish morpholino injection

ATG- and splice-blocking morpholinos were designed using Gene Tools’ oligo design service [73]. Resulting morpholino sequences were aligned against the zebrafish genome using BLAST [74] to ensure a lack of off-target binding. Morpholinos were re-suspended in sterile water to a concentration of 1 mM and diluted to working concentration with Danieau’s solution (58 mM NaCl, 0.7 mM KCl, 0.4 mM MgSO_4_, 0.6 mM Ca(NO_3_)_2_, 5 mM HEPES). Male and female wild-type adult zebrafish were housed and embryos bred using standard protocols [75]. Morpholinos were introduced into the zebrafish yolk via microinjection at no later than the two-cell developmental stage. Injected embryos were then kept at 28.5°C in E3 solution (5 mM NaCl, 0.17 mM KCl, 0.33 mM CaCl_2_, 0.33 mM MgSO_4_).

#### Measurement of cardiac output

Videos at 250 fps of beating hearts were taken from live 48 hours post-fertilization (hpf) embryos using an Axioplan (Zeiss) upright microscope with a 5× objective lens and a FastCam-PCI high-speed digital camera (Photron USA). In-house software (implemented in MATLAB [76]; available upon request) was used to determine heart rate from sequential image files, while measurements of ventricular long and short axis in both diastole and systole were obtained manually for each video using ImageJ [77] and used to estimate chamber volume using standard geometric assumptions. Cardiac output was then calculated as diastolic minus systolic ventricular volume multiplied by heart rate, as previously described [11].

#### Voltage mapping

Hearts were isolated from embryos at 48 hpf and stained with the transmembrane-potential-sensitive dye di-8-ANEPPS (Life Technologies, Carlsbad, CA, USA). Resulting fluorescence intensities were recorded with a high-speed charge-coupled-device camera (RedShirtImaging, Decatur, GA, USA) and images analyzed using software implemented in MATLAB [76]. For all comparisons regions of interest were determined and compared between morpholino injected embryos and controls from at least two separate mate pairings using a two-sided Student’s *t*-test.

#### Immunostaining

Hearts were isolated from embryos at 48 hpf and fixed using Prefer fixative (Anatech, Battle Creek, MI, USA). Fixed hearts were permeabilized using Phosphate Buffered Saline with Tween 20 and incubated overnight with a 1:200 diluted mouse anti-beta-catenin primary antibody (BD Biosciences, San Jose, CA, USA), then subsequently incubated with a 1:1,000 diluted donkey anti-mouse Alexa Fluor 488 conjugated secondary antibody (Life Technologies). Hearts were mounted using ProLong Gold Antifade Reagent with DAPI (Life Technologies) and imaged using a Leica SP5X laser scanning confocal microscope at 63× magnification. Resulting images were analyzed using ImageJ.

### Capture-based sequencing

NimbleGen’s SeqCap EZ Choice technology was used to construct oligonucleotide probes complementary to all exons (Ensembl GRCh37) of 116 genes involved or predicted to be involved in Mendelian forms of cardiac disease (Additional file 47). To identify established DCM candidates, we started from several recent reviews [39,71,72] of genes identified through family studies and updated these with any additional examples from the literature and commercial genetic testing panels. We included 13 additional candidates from our OPEN DCM candidates, primarily guided by interests in proteostasis and cardiac-specific splicing as well as three prioritized candidates from the LVD GWAS (*FLNC*, *USP13*, *SVIL*). Genomic DNA from 60 patients with idiopathic DCM enrolled in the Heart Failure Clinic at the University of California San Francisco was prepared using the Kapa Biosystems Library Preparation Kit, hybridized to the capture probes, and sequenced on an Illumina HiSeq 2000 sequencer. Multiplexing of 24 samples per lane of the sequencer flow cell yielded a median coverage of >700-fold, with fewer than 30 base pairs per sample having less than 30-fold coverage. Assembly was performed using *bwa* [78] and variants called using the *GATK* [79] (Additional file 48). Sanger sequencing was used to validate the *FLNC* splice site mutation. To assess the rate of *FLNC* mutations in controls, Variant Call Format files for the NHLBI Exome Sequencing Project [34] and 1000 Genomes Project [80] (1000 Genomes Project Consortium, 2010) were downloaded and annotated for functional consequence. The NHLBI exome data included two rare out-of-frame deletions (chr7:128498119 CTG > C and chr7:128498167 CCA > C) mapping to the carboxy-terminal 5% of the protein, a location of enrichment for common indels [81]. No similarly deleterious mutations were found in the 1000 Genomes data. Informed consent precludes re-contacting individuals for further study.

### Ethics

Human genetic studies were performed according to institutional guidelines and with the full approval of the University of California San Francisco Committee on Human Research (CHR #H1083-33104 and #10-00207) and all studies performed were in keeping with the original informed consent forms.

All zebrafish experimental work conforms to the ‘Guide for the Care and Use of Laboratory Animals’ published by the US National Institutes of Health (NIH Publication No. 85-23, revised 1996).

### Data deposition

Source code is available for download under a Creative Commons License at [82]. Sequencing data have been deposited in the Sequence Read Archive (SRA) under BioProject accession SRP049169.

OPEN predictions for GWA loci are available at http://cvri.ucsf.edu/~deo/disease_mapping.html.

## Additional files

Additional file 1: Table S1. The genes shared between Mendelian lipid disorders and GWA loci for lipid traits. Additional file 2: Table S2. Genes at the overlap of experimental evidence and GWA loci. Additional file 3: Table S3. OPEN performance metrics. Additional file 4: Table S8. OPEN predictions for height. Additional file 5: Table S9. OPEN predictions for rheumatoid arthritis. Additional file 6: Table S10. OPEN predictions for electrocardiogram phenotypes. Additional file 7: Table S11. OPEN predictions for QT interval. Additional file 8: Table S12. OPEN predictions for cardiac conduction phenotypes. Additional file 9: Table S13. OPEN predictions for platelet phenotypes. Additional file 10: Table S14. OPEN predictions for white blood cell phenotypes. Additional file 11: Table S15. OPEN predictions for leukemia. Additional file 12: Table S16. OPEN predictions for inflammatory bowel disease. Additional file 13: Table S17. OPEN predictions for asthma. Additional file 14: Table S18. OPEN predictions for uric acid levels. Additional file 15: Table S19. OPEN predictions for Alzheimer disease. Additional file 16: Table S20. OPEN predictions for celiac disease. Additional file 17: Table S21. OPEN predictions for cancer. Additional file 18: Table S22. OPEN predictions for systemic lupus erythematosus. Additional file 19: Table S23. OPEN predictions for breast cancer. Additional file 20: Table S24. OPEN predictions for colon cancer. Additional file 21: Table S25. OPEN predictions for high-density lipoprotein (HDL)-cholesterol. Additional file 22: Table S26. OPEN predictions for psoriasis. Additional file 23: Table S27. OPEN predictions for weight phenotypes. Additional file 24: Table S28. OPEN predictions for bone density. Additional file 25: Table S29. OPEN predictions for PR interval. Additional file 26: Table S30. OPEN predictions for chronic lymphocytic leukiemia. Additional file 27: Table S31. OPEN predictions for coronary artery disease. Additional file 28: Table S32. OPEN predictions for total cholesterol. Additional file 29: Table S33. OPEN predictions for type I diabetes mellitus. Additional file 30: Table S34. OPEN predictions for LDL-cholesterol. Additional file 31: Table S35. OPEN predictions for blood pressure phenotypes. Additional file 32: Table S36. OPEN predictions for triglycerides. Additional file 33: Table S37. OPEN predictions for multiple sclerosis. Additional file 34: Table S38. OPEN predictions for QRS duration. Additional file 35: Table S39. OPEN predictions for heart rate. Additional file 36: Table S40. OPEN predictions for C-reactive protein. Additional file 37: Table S41. OPEN predictions for glucose phenotypes. Additional file 38: Table S42. OPEN predictions for RBC and iron phenotypes. Additional file 39: Table S43. OPEN predictions for kidney function. Additional file 40: Table S44. OPEN predictions for primary biliary cirrhosis. Additional file 41: Table S45. OPEN predictions for fibrinogen. Additional file 42:
**Video.** Cardiac imaging of the *flnc* morphant. Additional file 43: Table S46. Phenotypes merged together into larger subsets for machine learning. Additional file 44: Table S4. Genes used for training for HCM and DCM predictions. Additional file 45: Table S7. Genes with nominally significant GWA loci for LVD. Additional file 46: Table S5. Genes implicated in Mendelian forms of cardiomyopathy. Additional file 47: Table S6. Genes whose exons are included on the targeted sequencing panel. Additional file 48: Table S47. Mutations in the 13 candidate DCM genes sequenced in DCM patients.

## Abbreviations

AUROC: area under the receiver operating characteristic curve
CMP: cardiomyopathy
CVD: cardiovascular disease
DCM: dilated cardiomyopathy
GBM: gradient boosting machine
GEO: Gene Expression Omnibus
GO: Gene Ontology
GWA: genome-wide association
HCM: hypertrophic cardiomyopathy
hpf: hours post-fertilization
kb: kilobase
LDL: low-density lipoprotein
LVD: left ventricular diameter
miRNA: microRNA
MMS: motif match score
MPD: Mouse Phenotype Database
OMIN: Online Mendelian Inheritance in Man
OPEN: Objective Prioritization for Enhanced Novelty
PWM: position weight matrix
ROC: receiver operating characteristic
SNP: single nucleotide polymorphism
TFBS: transcription factor binding site
